# Editorial: The multifaceted roles of lipids in physiological and pathophysiological states

**DOI:** 10.3389/fphys.2022.930962

**Published:** 2022-08-15

**Authors:** Simona Lobasso, Jean-Marc A. Lobaccaro, Roberto Angelini

**Affiliations:** ^1^ University of Bari Aldo Moro, Bari, Italy; ^2^ iGReD, Université Clermont Auvergne, CNRS6293, INSERMU1103, Clermont-Ferrand, France; ^3^ Swansea University Medical School, Swansea, United Kingdom

**Keywords:** lipid-metabolism, lipidomic analysis, physiopathogenesis, lipids, lipid homeostasis

Lipids have been first described during 18th century and since then they have been described in all living tissues. The chemistry of lipids started when the French chemist Poultier de la Salle (1719–1788) for the first time isolated cholesterol crystals from bile. Although Poultier’s work was never published, his disciples had quoted it later.

Recently, the advance in technology further improved the ability to characterize their highly variable structure and function. Almost 250 years later, this special volume of Frontiers in Physiology entitled “*The multifaceted roles of lipids in physiological and pathophysiological states*” aims to cover the last advances in lipid physiology. Because the human lipidome is made of thousands of lipid molecules, it is no wonder that their different chemical structures may exert an enormous variety of biological functions. These include energy production and membrane structural scaffold, sorting and regulation of membrane proteins, cellular signaling and vesicle trafficking. Lipidomics has been developed to study qualitatively and quantitatively these multifaceted molecules. This OMICS technology is a relatively young branch of analytical chemistry as well as an interdisciplinary field of study involving biochemistry and biophysics, applied mass spectrometry, complex statistical analyses, and miniaturization of the assays, which needs increased analytical standardization, especially in the quantification of individual lipid species. In addition, fine alterations of lipid composition can lead to a wide spectrum of human pathologies, ranging from cancer to metabolic, cardiovascular and neurodegenerative diseases. Enlightening this complexity, the various articles pinpoint some of these facts. The readers will thus find a Research Topic of articles on lipid functions ranging from oxysterols to cardiolipin, from fatty acids derivatives to protein lipidation and beyond.

Indeed, Wiley et al. describe new methods to assess activity of enzymes responsible for biosynthesis and degradation of endocannabinoid and related lipids in various mouse mucosal tissues. Griffiths and Wang suggest that oxysterols could rapidly act as a paracrine version of free cholesterol, mainly for resistance to microbial pathogens. Bozelli et al. enlighten the links between plasmalogens, common glycerophospholipids, and chronic inflammatory pathologies, and how they can be used to prevent inflammation. Engel et al. stress that determination of lipid biomarkers such as lysophosphatidylcholine needs a solid expertise. This point is crucial especially whether this biomarker is used to define pathological processes such as infertility, metabolic disorders, and cancers. Elkes et al. point out that diet supplementation with linoleic acid could ameliorate the effects of tafazzin deficiency in an animal model of the cardiomyopathy Barth syndrome. The link between lipids and immune cell physiology is ascertain by Zhang et al. work. It describes the lipid droplets as a “central hub” connecting metabolism and inflammation, and not only as lipid “bags”. Lobasso et al. report a comprehensive lipid analysis of exosomes secreted from melanoma cells having different metastatic behavior by a lipidomic approach. Hamsanathan and Gurkar review the current knowledge on lipid metabolism and cellular lipids during senescence. They clearly identify specific lipids such as 15d-PGJ2 as a biomarker of senescent cell removal (senolysis). The authors also define C16:0 ceramide level as a prognosis marker of functional decline. Likewise, Dai et al. describe that lifespan could be regulated by phosphatidylethanolamine, phosphatidylserine and cardiolipin levels, even though the precise mechanism is still unknown. Lipids may also be associated to proteins to modify their activity. This point is exemplified by Thomas et al. with Ghrelin, an orexigenic hormone that presents a unique octanoyl modification within its peptide sequence. The authors discuss the role of Ghrelin acylation for the brain physiology and the possible consequences of the acylation for cognition and neuropathology.

Ralph-Epps et al. summarise the important role of cardiolipin in Barth syndrome using the yeast model *Saccharomyces cerevisiae*. Beside an understandable alteration of the mitochondrial bioenergetics, the authors highlight that tafazzin defects also impair iron and calcium metabolism.

Altogether, this Research Topic, even though not exhaustive, offers various examples of successful strategies for dissecting out the multifaceted roles of lipids in physiology and pathology. We believe that this volume could be a springboard for other Research Topics that focus on lipid measurements, lipid homeostasis and their role in pathologies.

